# Solid Organ Transplant–associated Lymphocytic Choriomeningitis, United States, 2011

**DOI:** 10.3201/eid1808.120212

**Published:** 2012-08

**Authors:** Adam MacNeil, Ute Ströher, Eileen Farnon, Shelley Campbell, Deborah Cannon, Christopher D. Paddock, Clifton P. Drew, Matthew Kuehnert, Barbara Knust, Robert Gruenenfelder, Sherif R. Zaki, Pierre E. Rollin, Stuart T. Nichol, the LCMV Transplant Investigation Team

**Affiliations:** Centers for Disease Control and Prevention, Atlanta, Georgia, USA (A. MacNeil, U. Ströher, E. Farnon, S. Campbell, D. Cannon, C.D. Paddock, C.P. Drew, M. Kuehnert, B. Knust, S.R. Zaki, P.E. Rollin, S.T. Nichol);; and Arkansas Organ Recovery Agency, Little Rock, Arkansas, USA (R. Gruenenfelder)

**Keywords:** LCMV, lymphocytic choriomeningitis, organ transplant, transplant-associated infection, organ donation, viruses, zoonoses

## Abstract

Three clusters of organ transplant–associated lymphocytic choriomeningitis virus (LCMV) transmissions have been identified in the United States; 9 of 10 recipients died. In February 2011, we identified a fourth cluster of organ transplant–associated LCMV infections. Diabetic ketoacidosis developed in the organ donor in December 2010; she died with generalized brain edema after a short hospitalization. Both kidneys, liver, and lung were transplanted to 4 recipients; in all 4, severe posttransplant illness developed; 2 recipients died. Through multiple diagnostic methods, we identified LCMV infection in all persons, including in at least 1 sample from the donor and 4 recipients by reverse transcription PCR, and sequences of a 396-bp fragment of the large segment of the virus from all 5 persons were identical. In this cluster, all recipients developed severe illness, but 2 survived. LCMV infection should be considered as a possible cause of severe posttransplant illness.

## Abstract 2

Lymphocytic choriomeningitis virus (LCMV) is carried by rodents. In very rare instances, it has been transmitted from person-to-person by organ transplantation. In 2011, a total of 14 organ recipients were infected with the virus, of which 11 died in the United States. The 4 most recent patients received organs from the same donor, which resulted in 2 deaths. Only after these 4 organ recipients became sick was it discovered that the donor had been exposed to rodents. Had this exposure been known before transplantation, the organ recipients may have been more closely monitored. Early diagnosis and treatment might have improved their chances of survival. Although organ donor screening reduces the risk for transmission of some viruses, it is not possible to screen for all possible viruses, including LCMV. For patients who get severely ill after receiving a transplant, clinicians should add LCMV infection to their list of possible causes.

Lymphocytic choriomeningitis virus (LCMV), an Old World arenavirus, family *Arenaviridae*, is a zoonotic virus maintained in the house mouse (*Mus musculus*) and can be carried by pet and laboratory rodents (*1**–**7*); human exposure occurs through aerosolized excreta or by direct rodent contact. Infection in immunocompetent humans most commonly results in nonspecific febrile illness, although aseptic meningitis develops in a subset of persons (*8*). Person-to-person transmission of LCMV is unusual and has been reported only through vertical transmission from a pregnant woman to her fetus and through solid organ transplantation. In both instances, infections are associated severe disease. For instance, congenital infection can result in birth defects, including hydrocephalus and chorioretinitis (*9**–**12*), and transplant recipient infection can result in multisystem organ failure. Three previous clusters of organ transplant–transmitted LCMV infections have been identified in the United States, affecting 10 organ recipients, 9 of whom died (*13**,**14*).

In February 2011, the Centers for Disease Control and Prevention (CDC, Atlanta, GA, USA) was notified of a cluster of severe illnesses (2 fatal, and 2 in persons who were recovering) among 4 organ recipients linked to 1 donor, who died in late December 2010. Postmortem evaluation of the donor showed only evidence of previous Epstein-Barr virus infection. CDC acquired multiple specimens from the donor and recipients for testing. Histopathologic findings showed multifocal hepatocellular necrosis (Figure 1) in the lung transplant recipient, and Old World arenavirus antigens subsequently were identified by immunohistochemical testing (IHC). Reverse transcription PCR (RT-PCR) and sequencing indicated LCMV infection. Subsequent testing of specimens from the donor and recipients confirmed LCMV infection in all 5 persons, marking the fourth detected cluster of transplant-associated LCMV transmissions in the United States. We describe the laboratory investigation and clinical outcomes of this recent cluster of transplant-transmitted LCMV infections (Table 1).

**Figure 1:**
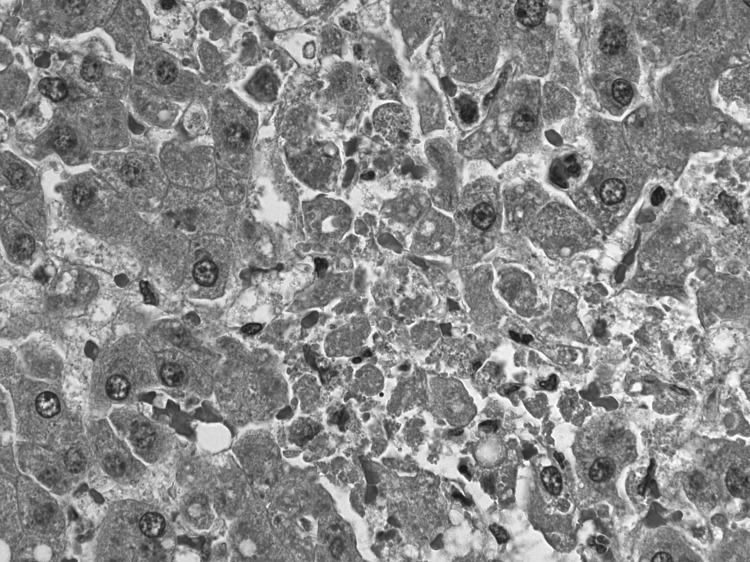
Liver from a 62-year-old woman (lung transplant patient) showing acute necrosis of hepatocytes and minimal inflammation. Randomly distributed single-cell necrosis, as observed in this patient, is a histopathologic feature observed in lymphocytic choriomeningitis virus infection. Original magnification ×400.

**Table 1:** Case information, major clinical findings, and outcome of an organ donor and 4 transplant recipients, United States, 2011*

Patient	Age, y	Organ received	Major clinical findings	Outcome
Donor	13	NA	Diabetic ketoacidosis, hypothermia, hypotension, nausea, vomiting, cerebral edema, possible meningitis	Died
Left kidney recipient	53	Left kidney	Urinary leak, pelvic abscess, acute respiratory distress syndrome, respiratory failure, mild hepatitis, possible acute myocardial infarction, possible encephalitis	Died
Right kidney recipient	46	Right kidney	Encephalitis, pancytopenia	Survived
Liver recipient	62	Liver	Hepatitis, encephalopathy, urinary tract infection, atrial fibrillation	Survived
Lung recipient	60	Lung	Pneumonia, respiratory failure, pulmonary infarction, atrial fibrillation, hepatitis	Died

*All were female. NA, not applicable.

## Methods

Medical teams involved in clinical care consulted with CDC. All available samples from the donor and the 4 recipients were then sent to CDC for diagnostic investigation.

### IHC

Tissue specimens were fixed in 10% neutral buffered formalin, embedded in paraffin, and cut into 4-μm sections. IHCs that used an immunoalkaline phosphatase technique (Fisc13) were performed on tissue sections. The primary monoclonal antibody (81001–52-BG12, Viral Special Pathogens Branch, CDC) reacts with the GP2 epitope of Lassa virus but also will react with other Old World arenaviruses, including LCMV. Appropriate positive and negative controls were run in parallel.

### RT-PCR

Total RNA was extracted from clinical specimens by using Tripure (Roche, Indianapolis, IN, USA) or from fixed tissue, as described (*15*). Because of the high genetic variability of LCMV (*16*), we used generic Old World arenavirus primers to amplify a 396-nt fragment from the large (L) segment (*17*). Negative samples underwent a second round of amplification with the same primers. The complete small (S) segment was amplified by previously described 19C primers (*18*). Resulting amplicons were purified and sequenced (GenBank accession nos. JN687949 [S segment] and JN687950 [L fragment]; because amplicon sequences from clinical samples were identical, a single sequence is provided for each amplicon). BLAST nucleotide analyses (http://blast.ncbi.nlm.nih.gov/Blast.cgi) were performed to verify the presence of LCMV.

### Serologic Testing

Postmortem serum from the donor, pretransplantation and/or posttransplantation serum from the recipients, and cerebrospinal fluid (CSF) collected from the right kidney recipient were sent to CDC. LCMV-specific IgM capture and IgG ELISA were performed as described (*19*).

## Results

### Clinical and Epidemiologic Investigations

#### Organ Donor

The organ donor, a 13-year-old girl with type 1 diabetes mellitus, was seen at an emergency department in December 2010 with a 2-day history of nausea and vomiting. At admission, her leukocyte count was elevated (19.1 × 10^3^ cells/μL, with 28% band forms and 56% segmented neutrophils (reference 3.54–9.07 × 10^3^ cells/μL, 0–5% bands, 40%–70% neutrophils]). Diabetic ketoacidosis was diagnosed and was managed with an insulin drip and aggressive fluid resuscitation. She reported a severe headache, which was not relieved by morphine. She vomited, aspirated, and required intubation and was noted to have muscle spasms and jerking before intubation. No lumbar puncture was performed. She became hypothermic and hypotensive requiring vasopressors; cerebral edema and coma developed, and the patient underwent emergency craniectomy, which was unsuccessful in preventing herniation. She was declared brain dead on day 2 of hospitalization. The family consented to organ donation, and her organs were procured the following day. Findings from autopsy indicated generalized brain edema; however, no evidence of an infectious or inflammatory process in the central nervous system (CNS) was noted. The left and right kidneys, liver, right lung, and corneas were procured and subsequently transplanted to 4 organ recipients and 1 cornea recipient (only 1 cornea was transplanted).

During the retrospective public health investigation, the girl’s residence was visited in April 2011. Family members reported that she had been sleeping in a recently built extension to the house and recalled rodent infestation in the extension when she became ill in December 2010. The family did not report having pet rodents or any other possible rodent exposures for the organ donor.

#### Left Kidney Recipient

This patient was a 52-year-old woman who underwent transplantation for end-stage renal disease caused by hypertension and diabetes mellitus. Her immunosuppressive regimen consisted of mycophenolate mofetil and tacrolimus. One week after transplantation, fever, nausea, vomiting, and diarrhea developed, and a urinary tract infection (UTI) was diagnosed. The fever was attributed to a pelvic abscess, which was drained, and a urinary leak was repaired. Three weeks after transplant, acute onset of severe headache and fever developed; acute respiratory distress syndrome also had developed, and the woman was intubated. Computed tomographic scan of the brain was unremarkable. Elevated cardiac enzymes and electrocardiographic changes also developed, as did anemia, thrombocytopenia (platelets 72 × 10^9^/L [reference 175–415 × 10^9^/L]), and mildly elevated transaminases (alanine aminotransferase [ALT] 74 U/L [reference 7–41 U/L]; aspartate aminotransferase [AST] 173 U/L [reference 12–38 U/L]). Her neurologic function was poor when sedation was reduced, but no lumbar puncture was performed because she had intermittent positional ventricular tachycardia and was too unstable for magnetic resonance imaging. Electroencephalography demonstrated background slowing. Supportive care was withdrawn, and the patient died 30 days after transplantation. No autopsy was performed.

#### Right Kidney Recipient

This patient was a 46-year-old woman who underwent transplantation for end-stage renal disease resulting from polycystic kidney disease. She received induction therapy with antithymocyte globulin and received maintenance immunosuppression with mycophenolate mofetil, tacrolimus, and prednisone; mycophenolate mofetil was later discontinued. Two days after transplantation, fever, which persisted during the following week; myalgia; severe headache; nausea; and vomiting developed. Lumbar puncture was performed 25 days after transplant; CSF contained elevated protein level (95 mg/dL [reference 15–50 mg/dL]), low glucose level (45 mg/dL [reference 10–70 mg/dL]), and increased leukocytes (188 cells/μL [reference 0 cells/μL]; 65% lymphocytes, 30% monocytes, and 5% segmented neutrophils). CSF cultures and PCR for herpes simplex virus and varicella zoster virus were negative. Aseptic meningitis was diagnosed, and the patient was treated with intravenous acyclovir, but no definitive cause of illness was identified. She was discharged 30 days after transplant but was readmitted 2 days later because of altered mental status; she had a 2-day history of nausea, vomiting, anorexia, and severe headache. CSF demonstrated elevated protein level (95 mg/dL), low glucose level (45 mg/dL), increased leukocytes (188 cells/μL; 65% lymphocytes, 30% monocytes, and 5% segmented neutrophils). Magnetic resonance imaging showed scattered multifocal signal abnormalities throughout the brain parenchyma. Pancytopenia developed, but extensive investigation for an infectious agent was unrevealing. She was treated again for possible herpes simplex encephalitis with intravenous acyclovir followed by oral valacyclovir, transferred to a rehabilitation facility 11 days later, and was discharged home the following week.

#### Liver Recipient

This patient was a 60-year-old woman who underwent transplantation for end-stage liver disease caused by alcoholic cirrhosis. She initially received an immunosuppressive regimen of mycophenolate mofetil, prograf, and prednisone; mycophenolate mofetil was later discontinued. Her postoperative course was complicated by UTI, postoperative encephalopathy, and elevated transaminases; liver biopsy showed no evidence of rejection. She was transferred to a rehabilitation facility but was readmitted 20 days after transplant with rapid atrial fibrillation and altered mental status. Hepatic transaminases were elevated (maximum ALT 366 U/L, maximum AST 564 U/L); liver biopsy 24 days after transplant demonstrated marked macrovesicular and microvesicular steatosis (>90%) and no evidence of rejection. Rapid atrial fibrillation was managed medically; pleural effusion developed, requiring thoracentesis. She had recurrent UTI, for which she received broad-spectrum antimicrobial drugs, and had *Clostridium difficile* colitis, which also was treated. She gradually recovered in a rehabilitation facility; liver enzymes normalized; and she was discharged home 66 days after transplant.

#### Lung Recipient

This patient was a 62-year-old woman who underwent lung transplantation for end-stage chronic obstructive pulmonary disease. She received 2 doses of basilixumab 0 and 4 days after transplant and a maintenance immunosuppressive regimen of mycophenolate mofetil, tacrolimus, and prednisone taper. She was extubated <1 day after transplant; however, rapid atrial fibrillation developed and was managed medically. She was discharged home after 1 week. Three days later, chills, dyspnea, fatigue, abdominal pain, nausea, and vomiting developed, and the patient’s oxygen requirement increased. She was readmitted 14 days after transplant; leukocyte count was 16,600 cells/μL; computed tomographic scan of the chest demonstrated pneumonia; and she was treated with broad-spectrum antimicrobial and antifungal drugs. Bronchoalveolar lavage culture grew *Candida albicans*. The patient was intubated 3 days after admission, after which fever, hypotension requiring vasopressors, and hepatitis (maximum ALT 744 U/L; maximum AST 1,133 U/L) developed. Asystole developed, and she died 6 days after being readmitted. Autopsy revealed ischemic cardiovascular disease, acute pulmonary infarction and necrosis of the right lower lobe, bilateral pulmonary edema and effusion, and extensive congestion of the liver.

#### Cornea Recipient

The cornea recipient was examined, as part of a follow-up visit, ≈4 months after transplant. She reported no illness after transplantation.

### Laboratory Investigation

We attempted to test all available samples remaining from the organ donor and recipients; for the liver recipient and the cornea recipient, we also were able to acquire follow-up serum samples. Diagnostic testing for LCMV was performed by using a combination of RT-PCR, serologic testing, and IHC (Table 2).

**Table 2:** Summary of laboratory test results for an organ donor and 4 transplant recipients, United States, 2011*

Patient	Time of sample collection	Specimen type	RT-PCR result	Serology	IHC
Organ donor	Postmortem	Serum	Neg	IgM neg, IgG neg	NA
	Postmortem	Autopsy tissues	Pos†	NA	Neg‡
	Postmortem	Cornea	Neg	NA	Neg
Left kidney recipient	11 d before transplant	Serum	Neg	IgM neg, IgG neg	NA
	22 d after transplant	Bronchoalveolar lavage	Pos	IgM neg, IgG neg	NA
Right kidney recipient	14 d before transplant	Serum	Neg	IgM neg, IgG neg	NA
	11 d after transplant	Bone marrow	Neg§	NA	Neg§
	32 d after transplant	CSF	Pos	IgM neg, IgG neg	NA
Liver recipient	7 d before transplant	Serum	ND	IgM neg, IgG neg	NA
	Before transplant	Liver	Neg	NA	Neg
	9 d after transplant	Liver	Pos	NA	Pos
	24 d after transplant	Serum	Pos¶	IgM pos (1,600), IgG neg	NA
	25 d after transplant	Liver	Pos	NA	Pos
	37 d after transplant	Liver	Pos	NA	Pos
	51 d after transplant	Serum	Neg	IgM pos (>6,400), IgG neg	NA
	98 d after transplant	Serum	Neg	IgM pos (>6,400), IgG neg	NA
Lung recipient	20 d after transplant	Autopsy tissues	Pos#	NA	Pos**
Cornea recipient	4 mo after transplant	Serum	ND	IgM neg, IgG neg	NA

*RT-PCR, reverse transcription PCR; IHC, immunohistochemical testing; neg, negative; NA, not applicable; pos, positive; CSF, cerebrospinal fluid; ND, not done. †Lymph node positive; spleen negative. ‡Central nervous system, spinal cord, trachea, lung, gastrointestinal tract, spleen, and mesenteric lymph node negative. §Bone marrow aspirate and biopsy specimens negative. ¶Virus was isolated from this specimen. #Lung and liver positive. **Bladder, pancreas, right lung, left lung, stomach, spleen, gall bladder, right adrenal gland, kidney, and liver positive; left ventricle negative.

#### Organ Donor

Serum, lymph node, and spleen were tested by RT-PCR for LCMV. The lymph node yielded a positive RT-PCR result when partial L segment primers were used. Sequencing of the RT-PCR–amplified fragment confirmed LCMV and identified a sequence different from any previously sequenced LCMV isolates in our laboratory. IgM capture and IgG ELISA on archived postmortem serum were negative for LCMV-specific antibodies. The nontransplanted cornea was also tested by IHC and RT-PCR; no evidence of viral antigen or RNA was detected in the cornea.

#### Left Kidney Recipient

Serum collected 11 days before transplant and tested for serologic evidence of previous LCMV infection was negative for LCMV-specific IgM and IgG, indicating that this patient had not been previously infected with LCMV; in addition, RT-PCR on this specimen was negative. Posttransplant specimens were limited to 1 bronchoalveolar lavage specimen collected 22 days after transplant. RT-PCR using L segment and full-length S segment primers gave positive results. Sequencing of both RT-PCR products indicated LCMV, and L segment product yielded a sequence identical to that from the organ donor. Phylogenetic analysis of the S segment indicated that the virus belonged to lineage I of LCMV (*16*).

#### Right Kidney Recipient

Serum collected 14 days before transplant tested negative for LCMV-specific IgM and IgG, indicating that this patient was not previously infected with LCMV; in addition, RT-PCR testing on this specimen was negative. CSF collected 32 days after transplant was positive for LMCV RNA by RT-PCR by using L segment and full-length S segment primers; sequencing of both products confirmed LCMV, and the L segment sequence was identical to that from the organ donor. The CSF specimen tested negative for LCMV-specific IgM and IgG.

#### Liver Recipient

**Figure 2:**
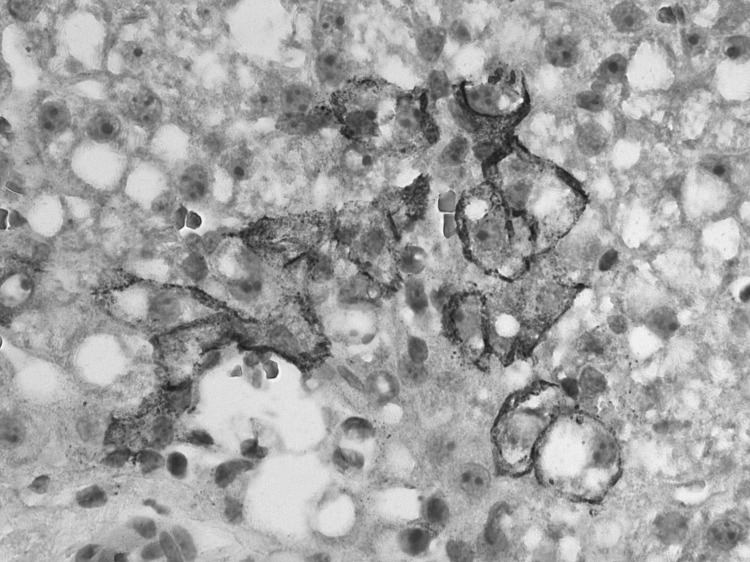
Immunohistochemical staining of lymphocytic choriomeningitis virus antigens in a biopsy specimen of the transplanted liver from a 60-year-old woman, which demonstrates abundant and predominantly perimembranous staining of hepatocytes. Original magnification ×200.

Liver biopsy specimens were acquired from the explanted native liver and the transplanted liver on days 9, 25, and 37 after transplant. The native liver tested negative for evidence of LCMV infection by RT-PCR, whereas all 3 posttransplant specimens yielded positive RT-PCR results (partial L segment primers); sequencing indicated a sequence identical to that from the organ donor. LCMV antigens also were detected by IHC in all 3 posttransplant liver biopsies (Figure 2) and absent in the native liver. Serum samples were acquired 7 days before transplant and 24, 51, and 98 days after transplant. The pretransplant serum sample was negative for LCMV-specific IgM and IgG, whereas posttransplant serum had LCMV-specific IgM (titers of 1,600, >6,400, and >6,400, respectively). No LCMV-specific IgG was detected in any of the serum samples. RT-PCR was performed on all serum samples; a positive result was obtained only for the serum collected 24 days after transplant, indicating that the virus was cleared from peripheral blood by day 51 after transplant. LCMV was additionally isolated from serum collected 24 days after transplant.

#### Lung Recipient

Lung and liver specimens collected at autopsy were RT-PCR positive by using partial L segment primers; sequencing of the amplified fragment indicated a sequence identical to that from the organ donor. Immunohistochemical evidence of LCMV infection was identified in numerous autopsy tissues, including bladder, pancreas, right lung, left lung, stomach, spleen, gall bladder, right adrenal gland, kidney, and liver.

#### Cornea Recipient

A serum sample collected ≈4 months after transplant from the cornea recipient tested negative for IgM and IgG. This finding indicated no evidence of previous LCMV infection.

## Discussion

We investigated a cluster of LCMV infections associated with organ transplantation. We found identical viral sequences in a 396-bp fragment of the L segment in clinical samples from the organ donor and all 4 organ recipients. Evidence of LCMV infection by IHC in tissues from 2 of the organ recipients supported this finding. Additionally, serum samples collected shortly before gorgan transplantation from 3 of the 4 recipients indicated absence of previous LCMV infection. Serologic testing of archived postmortem serum from the donor was negative for LCMV-specific antibodies, implying a relatively acute infection.

This event is the fourth cluster of organ transplant–transmitted LCMV infections identified in the United States and the fifth such transmission event reported worldwide (*13**,**14**,**20*). The previous 4 instances involved a total of 13 infected organ transplant recipients, 12 of whom died. Ribavirin is an antiviral drug with demonstrated efficacy for improving clinical outcomes for patients with Lassa virus (a closely related Old World arenavirus) infection (*21*). Although not definitely shown to have a therapeutic role in treating LCMV in humans, the 1 person who previously survived organ transplant–associated LCMV was treated with ribavirin (*13*). In this current cluster, 2 of the 4 infected recipients survived, without receiving therapy targeted at LCMV infection.

In immunocompetent persons, LCMV infection can cause CNS disease, such as aseptic meningitis. In previously documented LCMV infections associated with organ transplantation, a variety of signs and symptoms, including multisystem organ failure, developed in transplant recipients (*13**,**14*). Similarly, a diverse set of signs and symptoms were noted among recipients in this cluster, including evidence of meningoencephalitis in 3 recipients and the donor. Both persons who died had multisystem organ involvement: the left kidney recipient had acute respiratory distress syndrome and possible hepatic, cardiac, and CNS involvement, and the lung recipient had prominent pulmonary involvement and hepatitis. Severe pulmonary involvement was previously reported in lung recipients with transplant-transmitted LCMV (*13*). Finally, although LCMV transmission to caregivers has not been shown, the use of universal precautions for care of transplant recipients with suspected LCMV infection might be warranted, given the potential for high viral titers in body fluids of infected transplant recipients.

Multiple instances of rabies virus transmission associated with cornea transplantation have occurred (*22**–**24*), which provide at least some precedence for transmission of viruses through cornea transplantation. Fischer et al. (*13*) reported the absence of clinical evidence of infection in 2 recipients of corneas procured from the organ donor involved in the 2005 organ transplant–transmitted LCMV outbreak; however, no samples were available to definitively determine the absence of infection. The availability of a preserved, nontransplanted donor cornea, and follow-up serum from the recipient of the donor’s other cornea, enabled us to investigate the potential for LCMV transmission through cornea transplantation. We found no evidence of LCMV in the donor cornea, and serologic evaluation indicated absence of LCMV infection in the cornea recipient. Collectively these data suggest that the potential for LCMV transmission through cornea transmission is small, although we cannot conclusively exclude the theoretical transmission of LCMV through cornea transplantation.

The absence of LCMV-specific IgG in the samples from the liver transplant recipient (including a sample 98 days after transplant) is noteworthy. These observations are consistent with those for the only previous surviving patient, who remained negative for LCMV-specific IgG 85 days after transplant (*13*). Although the availability and timing of clinical sample collection from persons with fatal outcomes from this and previous transplant clusters vary widely, no LCMV-specific IgG has been identified in any patients with fatal transplant-associated LCMV infections.

Although organ donation screening procedures reduce the risk for transmission of some bloodborne viruses, such as HIV, hepatitis B virus, and hepatitis C virus, through the donor history questionnaire and laboratory screening (*25**,**26*), screening for all possible acute viral infections from donor evaluation to organ procurement is not possible. Other notable instances of transplant-transmitted viral encephalitis from West Nile virus and rabies virus have occurred recently (*27**–**29*). Similarly, LCMV is not among the infectious agents routinely screened for in potential organ donors. However, for the cluster reported here, archived postmortem donor serum was negative for LCMV by RT-PCR and serologic testing, and thus testing serum before organ transplantation would not have helped recognize donor infection. Asking about exposure to rodents also might be helpful in heightening suspicion for LCMV infection in potential donors who have signs of aseptic meningitis. Although family members described clear evidence of rodent exposure for the organ donor shortly before onset of illness, this information was acquired during follow-up investigations, which occurred long after LCMV infection in the organ recipients. Current Organ Procurement and Transplantation Network guidelines require assessment of the donor’s medical history and behavior, including a review of the donor’s medical records (optn.transplant.hrsa.gov/policiesAndBylaws/policies.asp) before transplantation. Although recent rodent exposure by a potential organ donor would not exclude transplantation, the information might help transplant centers appropriately assess risk to the potential donor, heighten their suspicion for transplant-transmitted LCMV in the event of recipient illness, and obtain early diagnosis and treatment. The efficacy of ribavirin for treating LCMV infection in humans has not been examined; however, early detection of LCMV infection and treatment with ribavirin might improve the outcomes of transplant recipients with transplant-transmitted LCMV infection.
